# Climate and Health Co-Benefits in Low-Income Countries: A Case Study of Carbon Financed Water Filters in Kenya and a Call for Independent Monitoring

**DOI:** 10.1289/EHP342

**Published:** 2016-09-16

**Authors:** Amy J. Pickering, Benjamin F. Arnold, Holly N. Dentz, John M. Colford, Clair Null

**Affiliations:** 1 Civil and Environmental Engineering, Stanford University, Stanford, California, USA; 2 Center for Innovation in Global Health, Stanford University, Stanford, California, USA; 3 Division of Epidemiology, School of Public Health, University of California, Berkeley, Berkeley, California, USA; 4 Department of Nutrition, University of California, Davis, Davis, California, USA; 5 Innovations for Poverty Action, Nairobi, Kenya; 6 Mathematica Policy Research, Washington, DC, USA

## Abstract

**Background:**

The recent global climate agreement in Paris aims to mitigate greenhouse gas emissions while fostering sustainable development and establishes an international trading mechanism to meet this goal. Currently, carbon offset program implementers are allowed to collect their own monitoring data to determine the number of carbon credits to be awarded.

**Objectives:**

We summarize reasons for mandating independent monitoring of greenhouse gas emission reduction projects. In support of our policy recommendations, we describe a case study of a program designed to earn carbon credits by distributing almost one million drinking water filters in rural Kenya to avert the use of fuel for boiling water. We compare results from an assessment conducted by our research team in the program area among households with pregnant women or caregivers in rural villages with low piped water access with the reported program monitoring data and discuss the implications.

**Discussion:**

Our assessment in Kenya found lower levels of household water filter usage than the internal program monitoring reported estimates used to determine carbon credits; we found 19% (*n* = 4,041) of households reported filter usage 2–3 years after filter distribution compared to the program stated usage rate of 81% (*n* = 14,988) 2.7 years after filter distribution. Although carbon financing could be a financially sustainable approach to scale up water treatment and improve health in low-income settings, these results suggest program effectiveness will remain uncertain in the absence of requiring monitoring data be collected by third-party organizations.

**Conclusion:**

Independent monitoring should be a key requirement for carbon credit verification in future international carbon trading mechanisms to ensure programs achieve benefits in line with sustainable development goals.

**Citation:**

Pickering AJ, Arnold BF, Dentz HN, Colford JM Jr., Null C. 2017. Climate and health co-benefits in low-income countries: a case study of carbon financed water filters in Kenya and a call for independent monitoring. Environ Health Perspect 125:278–283; http://dx.doi.org/10.1289/EHP342

## Introduction

The recent Paris Agreement, which was signed by nearly 200 nations, aims to prevent global warming from exceeding 2°C above pre-industrial levels (UNFCCC 2015). The agreement acknowledges global disparities in present and historic energy consumption by including goals of sustainable development and poverty eradication alongside its primary goal of mitigating global greenhouse gas emissions. To enable countries to cost effectively meet their nationally determined emission reduction goals, the Paris Agreement also allows parties (e.g., countries) to transfer mitigation outcomes that help them achieve these goals and establishes an international mechanism to promote mitigation and support sustainable development (Article 6) (UNFCCC 2015). Predating the Paris Agreement, the clean development mechanism (CDM) created by the 1997 Kyoto Protocol (United Nations 1998) facilitated carbon offset projects in lower-income countries. The CDM was designed to reduce carbon emissions more cost effectively by allowing developed countries with emission reduction targets to purchase carbon credits from developing countries where projects cost less to implement. Similar to the Paris Agreement, a second explicitly stated goal of the CDM was to assist developing countries achieve sustainable development (United Nations 1998). A voluntary carbon market also exists outside of the United Nations Framework Convention on Climate Change and its legal instruments, in which corporations, organizations, and individuals typically purchase carbon credits to offset their emissions. Designated Operational Entities (DOE) serve as third-party auditors to validate CDM project proposals and certify carbon emission reductions (UNFCCC 2014). However, in both compliance and voluntary markets, carbon offset program implementers are allowed to collect their own monitoring data to submit to third-party certification agencies to determine the number of carbon credits awarded (UNFCCC 2014; http://www.goldstandard.org). Implementers have financial incentives to report high offsets in order to claim more credits, which could lead to biased results. In this commentary, we argue for the need to independently monitor emissions reduction programs, and we present a case study of a carbon offset project designed to provide safe drinking water in rural Kenya.

### Carbon Credits, Water Treatment, and Suppressed Demand

Carbon finance could be a financially sustainable approach to scale up water treatment and improve health in low-income settings, and in doing so would reduce greenhouse gas emissions while contributing to sustainable development (Hodge and Clasen 2014). Almost half (42%) of the global population does not have access to piped water into the home (WHO-UNICEF 2015), and it is estimated that 1.8 billion people drink from a water source with fecal contamination (Bain et al. 2014). Diarrhea is a leading cause of child mortality, causing 700,000 child deaths annually (Walker et al. 2013). Disinfection of drinking water supplies can prevent the transmission of common diarrheal pathogens (Prüss et al. 2002) and historically led to dramatic reductions in mortality associated with waterborne illness in the United States and Europe (Cutler and Miller 2005; Sedgwick and MacNutt 1910).

A number of programs to reduce greenhouse gas emissions are implementing zero-emission household water treatment technologies with carbon credit financing. Carbon credits (awarded for the avoidance, sequestration, or reduction of 1 ton of carbon dioxide equivalent) are generated by calculating the quantity of greenhouse gas emissions avoided by treating water with a zero-emission technology (e.g., water filter) against an estimated baseline of emissions if households had boiled the water using fossil fuel or nonrenewable biomass (Hodge and Clasen 2014). Greenhouse gas emissions resulting from the manufacturing, distribution, and behavior promotion of such water treatment technologies are also accounted for in carbon credit calculations. Baseline emissions can adjust upwards to account for “suppressed demand,” based on the concept that current emissions are constrained by limited resources in developing economies. For example, the baseline would include the carbon emissions theoretically emitted from households that would boil their drinking water if they had access to sufficient fuel and the resources to obtain it (independent of whether households actually boil their water). In addition to baseline emission estimates, a key parameter for awarding carbon credits under this system is the percentage of households that regularly use the water treatment units, determined by internal monitoring by the program implementer. Proponents emphasize the primary benefit of such programs is to improve water quality for high need populations; however, critics note greenhouse gas emissions are not actually reduced because most households would not boil their water in the absence of the programs (Yeo 2013).

### Carbon for Water in Western Kenya

During April and May of 2011, Vestergaard Frandsen reported that they distributed 877,505 LifeStraw® Family water filters free of charge to > 4.5 million people in Kenya’s Western Province (Vestergaard Frandsen 2012). The program is registered to earn carbon credits certified by a third-party organization, the Gold Standard Foundation (http://www.goldstandard.org), for use in the voluntary carbon market with a crediting period of 10 years. In this “Carbon for Water” program, all households received a LifeStraw® Family water filter [model 1.0; Vestergaard, http://www.vestergaard.com/lifestraw-family-1-0), a point-of-use water treatment product that does not require electricity to operate and is classified as “Highly Protective” by World Health Organization testing guidelines (WHO 2011). The Carbon for Water program implemented three household health education campaigns (July–August 2011, April–May 2012, and October 2012), employing almost 2,000 community health workers to personally visit households that had received a filter, and broadcasted messages about the program over the radio. The health messaging included training on proper filter use, as well as promotion of safe water storage to prevent recontamination, and handwashing with soap and filtered water. The program also established 32 complementary maintenance, repair, and education facilities in Western Province (Vestergaard Frandsen 2012). Vestergaard Frandsen hired staff to collect their own monitoring data (sampling between approximately 15,000 and 20,000 households each round), as well as contracted a local Kenyan firm to conduct audits (100–300 households each round). The program reported that it distributed filters to 91% of all households in Kenya’s Western Province and that water filter usage rates over time since distribution were 91% (0–6 months), 75% (7–18 months), and 81% (19–32 months) (Vestergaard Frandsen 2012, 2013, 2014a). The program assumed that 79.6% of households would boil their water if they had access to adequate resources in monitoring period 1 (0–6 months) and monitoring period 2 (7–18 months); this assumption was lowered to 52.8% of households for monitoring period 3 (19–32 months). The usage rates combined with the baseline emissions estimated using suppressed demand earned a total of 4,476,205 carbon credits during the period 1 June 2011–31 January 2014 (32 months) (Vestergaard Frandsen 2014a). Due to fluctuations in the price of carbon credits and unreleased implementation costs, actual program profits (or losses) to date are unknown.

## Methods

**Figure 1:**
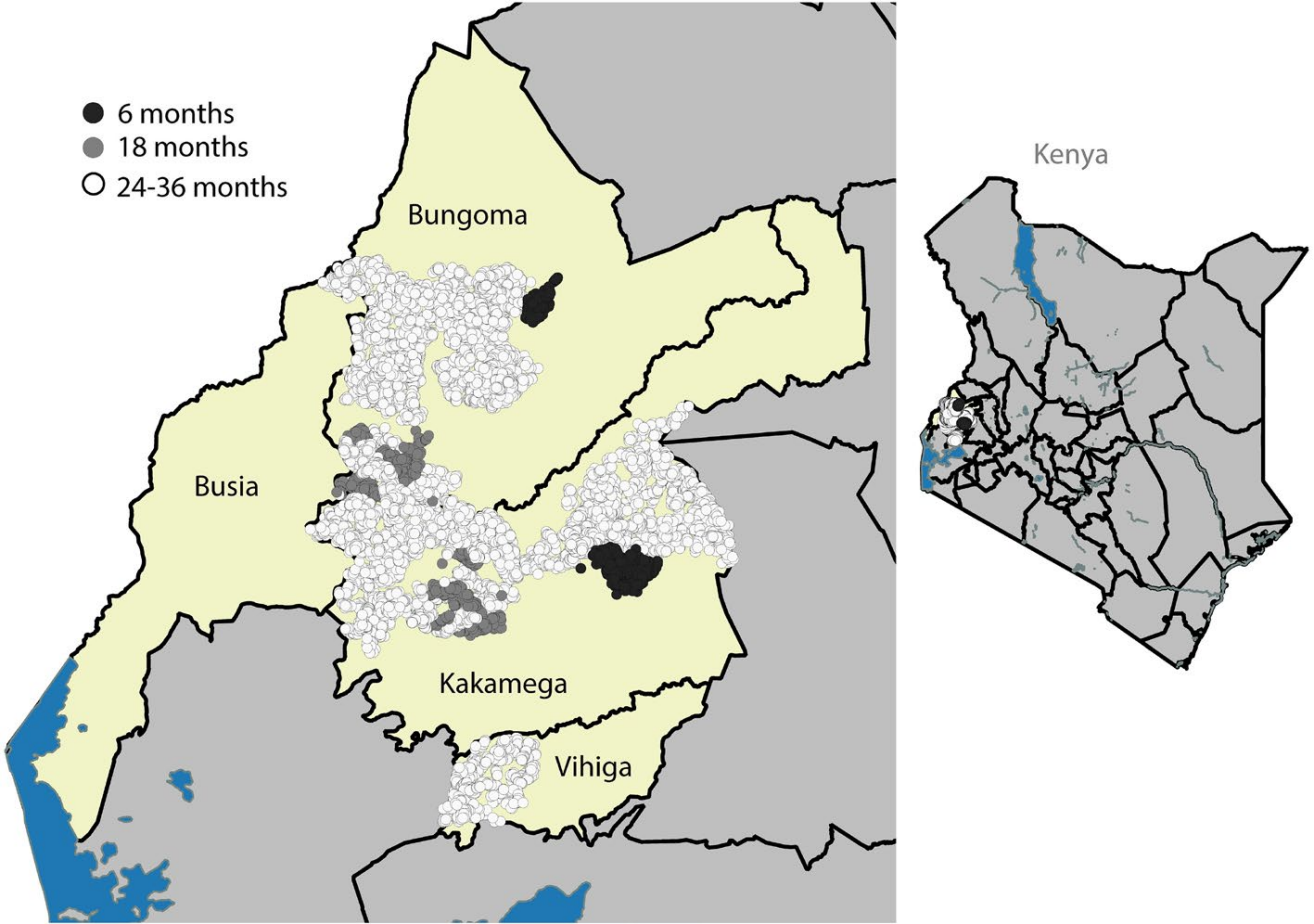
The Carbon for Water program distribution area is shown in light yellow (includes Bungoma, Kakamega, Vihiga, and Busia counties in Western Kenya). Each circle represents the location of one household enrolled in our assessment (*n* = 8,721); dark gray circles indicate study population A (surveyed approximately 6 months after LifeStraw^®^ filter distribution), light gray circles indicate study population B (surveyed approximately 18 months post filter distribution), open circles indicate study population C (surveyed approximately 24–36 months post filter distribution). The location of the study area within Kenya is shown at right.

The Kenya Carbon for Water program happened to overlap a large randomized controlled trial run by our research team evaluating the health effects of water, sanitation, hygiene, and nutritional interventions among newborns in rural villages in Kakamega, Bungoma, and Vihiga counties in Western Province (WASH Benefits study). As part of the WASH Benefits study, we collected household survey data to pilot test interventions and characterize baseline water management practices and drinking water quality in the WASH Benefits study population (Arnold et al. 2013; Christensen et al. 2015). The coincidental temporal and geographic overlap of WASH Benefits with the Carbon for Water program allowed us to measure household filter usage and microbial water quality over a 3-year period that included the first 32 months of program monitoring. Because of the potential importance of filter usage rates in interpreting the primary outcomes of the WASH Benefits trial, we added measures of filter ownership to existing household surveys that took place approximately 6, 18, and 24–36 months after the filter distribution by Vestergaard Frandsen during April and May in 2011. We sampled a different study population during each time period (Table 1). All surveys contained a standardized module to assess household water management practices, use of the LifeStraw® Family filter, and reasons for nonuse. Figure 1 is a map of household survey locations within the program area.

**Table 1:** Self-reported ownership and usage of LifeStraw^®^ Family filters among three study populations (A, B, and C) at 6, 18, and 24–36 months post-filter distribution and assessment of filter use based on observations by study staff.

Indicator	Population A 6 months	Population B 18 months	Population C 24–36 months
All households
otal number	499	531	7,691
Received filter, *n* (%)	453 (90.8)	374 (70.4)	4,041 (52.5)
Stored water not currently available, *n* (%)	46 (10.8)	59 (11.1)	1,485 (19.3)
Filtered currently stored water, *n* (%)	119 (26.7)	95 (20.1)	252 (4.1)
Uses filter as water treatment method, *n *(%)	281 (56.3)	201 (37.9)	792 (10.3)
Households that received filter
otal number	453	374	4,041
Stored water not currently available, *n* (%)	45 (9.9)	30 (8.0)	658 (16.3)
Filtered currently stored water, *n* (%)	118 (28.9)	93 (27.0)	236 (7.0)
Uses filter as water treatment method, *n *(%)	278 (61.4)	195 (52.1)	752 (18.6)
Could produce filter for observation, *n* (%)	421 (92.9)	354 (96.7)	3,616 (93.6)
Promotion visit by program in past 6 months	N/A	356 (95.2)	2,204 (54.6)
Missing		0	3
Households that produced filter
otal number	421	354	3,616
Filter not working	20 (4.8)	77 (21.8)	1,839 (51.0)
Don’t know	0	0	8
Filter hanging on wall^*a*^	393 (93.4)	340 (96.9)	3,360 (93.8)
Observation not possible	0	3	33
Moisture in filter reservoir^*a*^	136 (32.3)	89 (25.2)	438 (12.2)
Observation not possible	0	1	18
Filter has signs of nonuse (e.g., dust)^*a*^	299 (71.0)	228 (64.8)	2,963 (82.3)
Observation not possible	0	2	17
Note: Population A included households with pregnant women and caregivers of young children surveyed in November 2011, approximately 6 months after LifeStraw^®^ filters were distributed. Population B included households with pregnant women surveyed in November 2012, approximately 18 months after LifeStraw^®^ filters were distributed. Population C included households with pregnant women surveyed June 2013–May 2014, approximately 24–36 months after LifeStraw^®^ filters were distributed. ^***a***^Direct observation by field staff.			

The 6-month survey (1–29 November 2011) draws on data collected during the baseline assessment for two pilot randomized controlled trials conducted as part of WASH Benefits, simultaneously implemented in 72 rural villages in Western Kenya; the trials evaluated the adoption of household water, sanitation, and hand hygiene interventions [see Christensen et al. (2015) for further details]. These trials enrolled households with caregivers of 4- to 16-month-old children in Kakamega (367 households in the Shianda Location), and pregnant women and caregivers with children < 3 months near the town of Bungoma (132 households in the Kibingei Location). The 18-month survey (27 November–19 December 2012) and the 24–36 month survey (19 June 2013–21 May 2014) data were obtained from the baseline assessment and enrollment survey of the full-scale WASH Benefits study (Arnold et al. 2013). The WASH Benefits study enrolled pregnant mothers in their second or third trimester in rural villages with low levels of piped water access (< 20% of households) in Kakamega, Bungoma, and Vihiga counties. All respondents provided written informed consent; those not comfortable signing their name provided a thumb print. Human subjects institutional review boards (IRBs) at the University of California, Berkeley, Stanford University, and the Kenya Medical Research Institute (KEMRI) approved the study protocols.

Field staff asked respondents to fetch a cup of water the way they normally would for a young child, then observed from where the respondent obtained the water and how it was stored and extracted. Field staff inquired if anyone in the household “had done anything to make the water less cloudy or safer to drink,” and if so, what method was used (without prompting on specific water treatment methods). If the respondent did not report a water treatment method, the field staff asked if the respondent ever treats drinking water and to list all methods used. Field staff questioned if the household had received a LifeStraw® Family filter. If the household reported receiving a filter, we observed if the filter was present, hanging on the wall, looked unused (e.g., visible dust), and contained water or moisture. Field staff asked if the filter was working and if there were any issues that prevented use. Finally, respondents reported if and when a representative from the Carbon for Water program had most recently visited their home to promote the LifeStraw® filter.

We also collected a sample of stored drinking water to assess levels of *Escherichia coli* contamination during the 18-month and 24–36 month surveys, which allowed us to compare water that respondents indicated was filtered with a LifeStraw® filter to water reported by the respondent to be unfiltered. Respondents were asked to pour the fetched cup of stored water into a sterile 100 mL Whirl-Pak® bag (product no. B01040WA; Nasco, https://www.enasco.com/page/contact). The samples were placed on ice, transported to a field lab, and processed by membrane filtration within 8 hr. A volume of 100 mL was vacuum filtered, plated on MI agar, then incubated at 35°C for 24 hr. *E. coli* were enumerated following the U.S. Environmental Protection Agency (EPA) method 1604 (U.S. EPA 2002).

## Results

**Figure 2:**
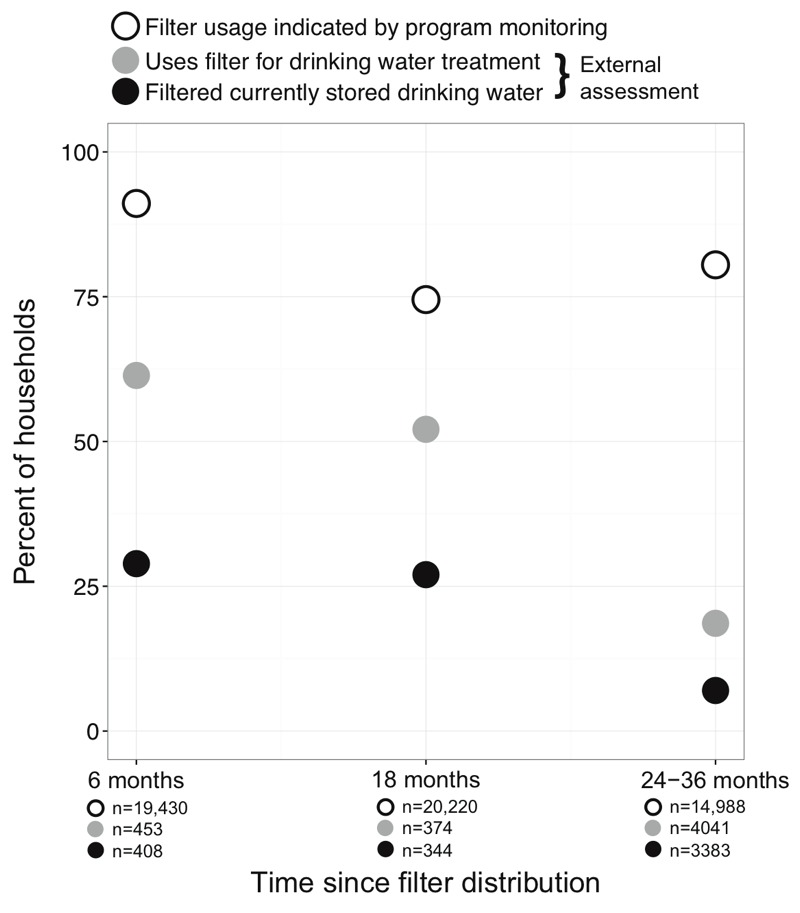
Self reported indicators of LifeStraw^®^ Family filter usage (solid black and gray circles) collected by our assessment of rural households with pregnant women or caregivers of young children that reported receiving a filter in Western Kenya; a separate study population was measured at each time point (6 months, 18 months, and 24–36 months). Open circles indicate reported usage in official program monitoring reports for carbon credit verification; the three monitoring periods ended at 6 months, 19 months, and 32 months post filter distribution. Standard errors (not shown) are less than 3 percentage points for all data points.

We completed 499 household surveys 6 months after filter distribution (study population “A” in Table 1), 531 household surveys 18 months after filter distribution (study population “B”), and 7,691 household surveys 24–26 months after filter distribution (study population “C”) as part of our on-going enrollment into the WASH Benefits pilot study and main study. Our measurements indicate that the Carbon for Water program coverage was impressively high; the percentage of households that remembered ever receiving a filter was 91% 6 months after filter distribution. However, this number fell to 70% at 18 months and 53% at 24–36 months (Table 1). Among households that reported filter ownership, 95% said the Carbon for Water program had visited their home to promote the LifeStraw® at the 18-month survey and 55% at 24–36 months (Table 1, this question was not asked at the 6-month visit). Although we did not measure the same households at each time point, we documented a progressive decline in reported filtering of currently stored drinking water among households that had received a filter: 29% at 6 months (118 out of 408 in study population A), 27% at 18 months (93 out of 344 in study population B) and 7% at 24–36 months (236 out of 3,383 in study population C) (Figure 2, Table 1). Similarly, the percentage of households that reported using the filter as a drinking water treatment method was lowest among study population C, and highest among study population A (Figure 2, Table 1). The percentage of filters with observed moisture or water (one indicator of recent use) also declined with time since distribution (32% at 6-months, 25% at 12-months, and 12% at 24–36 months). Half (51%) of households reported filters were not working after 24–36 months. When asked about issues preventing use of the LifeStraw® filter, 35% of households that received a filter said the filter was too slow or took too much time, 17% said the filter was blocked or not working, 8% said the filter had a bad smell or taste, and 7% thought the filter was bad for their health (data combined from all study populations, *n* = 4,868).

Among our full study population (all households in study populations A, B, and C), for drinking water most households access protected springs (52%, *n* = 7,170) or unprotected (16%) springs, 16% access shallow wells, with the remainder accessing borewells, surface water, or collecting rainwater; 71% (*n* = 7,170) of households access improved water sources according to the WHO/UNICEF Joint Monitoring Program definition (WHO-UNICEF 2016). The percentage of households accessing improved water sources is similar to the 68% reported in the Carbon for Water program’s third monitoring report (Vestergaard Frandsen 2014a), as well as the 67% reported by the 2014 Demographic and Health Survey (DHS Program 2015). Among our full study population, filtering of stored drinking water was not significantly different between households accessing improved water sources versus those accessing unimproved water sources (6.6% vs. 6.4%; *p* = 0.786). Among all households in our study with available stored water, field staff observed the majority (78%, *n* = 7,041) of stored drinking water containers to be covered. When asked to retrieve a glass of water, 61% (*n* = 7,069) of respondents extracted water by dipping a cup into the storage container.

Microbial water quality was improved in filtered stored drinking water we collected from our study populations B and C (measured 18 months and 24–26 months after filter distribution), but typically still reflected contamination with the fecal indicator bacteria *Escherichia coli.* The geometric mean in filtered water was 20.6 *E. coli* per 100 mL (*n* = 296), compared to 27.5 *E. coli* (*n* = 5,492) in unfiltered water (*p* = 0.012). Over half (52%) of respondents reported storing filtered water for 2 or more days, indicating the potential for recontamination during storage. Water treatment methods other than filter usage cited by respondents included adding chlorine (5%, *n* = 7,122) and boiling (1%, *n* = 7,122) (data from all three study populations). Stored water reported by households to be treated with locally available chlorine had a geometric mean < 10 *E. coli* per 100 mL (*n* = 311 samples from study populations B and C).

## Discussion

Our measurements show lower usage of LifeStraw® Family filters compared with the program’s own monitoring data (Figure 2). We document 19% usage of the filters as a drinking water treatment method 2–3 years after filter distribution (among households with pregnant women) compared to the 81% usage reported by the program approximately 2.7 years after filter distribution (Vestergaard Frandsen 2014a). In addition, while data reported by the Carbon for Water program indicated consistent high use over time, our data suggest that use decreased over time. These findings suggest that providing water treatment technologies like the Lifestraw® Family filter do not automatically translate into improved water quality at the point of use (and by extension improved health). This result is consistent with other studies showing poor long-term adoption and inconsistent use of household water treatment products provided programmatically (Arnold et al. 2009; Luby et al. 2008; Rosa et al. 2016). A large systematic review found no evidence that household water treatment interventions reduce child diarrhea after 12 or more months of implementation, possibly because product usage is not sustained (Clasen et al. 2015). Considering the equivocal success of previous household water treatment programs at achieving sustained access to safe water, future greenhouse gas reduction programs may want to consider implementing emission-free water treatment technologies that automatically treat drinking water at the community level instead of relying on users to consistently treat their own water (Amin et al. 2016; Pickering et al. 2015).

Providing access to safe water in Kenya is a stated goal of the Vestergaard Frandsen Carbon for Water program (Vestergaard Frandsen 2014b). The immense scale of such programs presents enormous opportunity to improve water quality for millions living in poverty. We believe greenhouse gas emissions reduction programs claiming to provide safe water to low-income people should be required to demonstrate that improvements in drinking water quality are actually achieved and sustained though regular independent monitoring of microbial water quality. We found low prevalence of boiling (1%) to treat drinking water by households with and without a LifeStraw® Family filter, indicating the Carbon for Water program in Kenya has achieved minimal reductions in actual greenhouse gas emissions (in contrast with the projected reductions from hypothetical baseline emission levels estimated using suppressed demand). When suppressed demand is employed to calculate baseline scenarios, the potential absence of actual greenhouse gas emissions reductions places additional onus on programs to improve sustainable development outcomes, such as drinking water quality.

## Conclusion

The 2015 Paris Agreement provides a timely opportunity to create a new or updated international trading mechanism that could improve the environmental, health, and economic benefits of future emissions reduction programs by mandating independent monitoring for carbon credit verification. Furthermore, carbon offset programs will only contribute to the Paris Agreement’s goals of greenhouse gas emissions reduction, sustainable development, and poverty alleviation if they are implemented successfully. Without independent evaluation, it is difficult to confirm that monitoring results are accurate. Evaluations of international development programs are widely acknowledged to be stronger if conducted independently from the implementing organizations because of the inherent conflict of interest—financial and otherwise—that implementers have in the success of their own programs (Gertler et al. 2011; Purcell 2003; Savedoff et al. 2006; USAID 2016). Similar to monitoring guidelines for clinical research studies and finances of publicly traded companies, we propose that carbon credit program monitoring standards be revised to ensure that monitoring activities are free from real or perceived conflicts of interest.

One strategy for achieving independent cost-effective monitoring would be to transfer funds that program implementers already spend on their own internal monitoring to the third-party credit certification organizations, which in turn would contract independent evaluations. This would expand the scope of credit certification organizations, but would help prevent conflicts of interest arising in the evaluation process. The monitoring process would also ideally include pre-specified indicators used to evaluate non-emissions related benefits (Miguel et al. 2014), such as microbial water quality for water treatment projects (Hodge and Clasen 2014). In addition, a reporting schedule tied to publicly available results would facilitate prompt feedback to program implementers to improve program effectiveness, improve transparency to the global community, and improve data reliability.
